# O-09 EVALUATING PERFORMANCE OF GROUP A STREPTOCOCCAL LATERAL FLOW DEVICES IN CARE HOME OUTBREAKS IN ENGLAND: A PILOT STUDY, AUTUMN 2025

**DOI:** 10.1093/pubmed/fdag046.009

**Published:** 2026-07-09

**Authors:** Ms Eve Matthews, Joshua Carter, Clare Sawyer, Carmellie Inzoungou-Massanga, Motolani Awokoya, Neil Bray, Oluwakemi Olufon, Rohini Manuel, Theresa Lamagni, Obaghe Edeghere, Deepti Kumar

**Affiliations:** Field Service Rapid Investigation Team, UK Health Security Agency; Field Service Rapid Investigation Team, UK Health Security Agency; Field Service South East and London; Field Service Rapid Investigation Team, UK Health Security Agency; Field Service Rapid Investigation Team, UK Health Security Agency; Field Service Rapid Investigation Team, UK Health Security Agency; Field Service Rapid Investigation Team, UK Health Security Agency; Field Service South East and London; Antimicrobial Resistance & Healthcare-Associated Infection Division; National Field Services; Field Service Rapid Investigation Team, UK Health Security Agency

## Abstract

**Background:**

Group A *Streptococcus* (GAS) outbreaks in care homes lead to significant morbidity among residents. In 2025, 36 iGAS care home outbreaks were reported in England, throat swabs for culture were collected in 56% of these. Lateral flow devices (LFDs) offer rapid GAS detection for prompt initiation of control measures. LFD sensitivity and specificity for detecting asymptomatic carriage is unknown.

**Objectives:**

We aimed to test feasibility and methodology to evaluate the diagnostic accuracy and acceptability of LFDs in GAS care home outbreaks.

**Methods:**

We took throat swabs for LFD and culture from staff and residents, as directed by the incident management team for an iGAS outbreak in a care home. We compared the diagnostic accuracy of LFDs (index test) to culture (reference standard). We asked staff about acceptability and recorded result turn-around time and operational feasibility. LFD results weren’t shared outside the study team.

**Results:**

We recruited 80 residents and staff. Amongst those approached, the uptake rate was 97.5% overall, 98% for staff (60/61) and 95% for residents (20/21). Individual results were available within 5 minutes for LFDs and between 2-4 days (median 3) for culture. All LFD and culture results were concordant and negative. Based on 80 culture negative participants, specificity is estimated at 100% (95.4%-100%) in this setting.

Amongst healthcare staff, 96% (46/48) would hypothetically be willing to perform an LFD self-test and 85% (41/48)​ on residents; 98% (47/48) would feel confident hypothetically reading the result of an LFD.

**Conclusions:**

GAS LFDs are likely to be highly specific; we report an estimate with a relatively few non-infected participants and cannot estimate sensitivity. GAS LFDs provide results substantially quicker than culture and are highly acceptable to staff within a care home setting. A full prospective evaluation of LFD use in care homes is needed to inform management of GAS outbreaks in care homes.

